# Epithelioid Sarcoma of the Oral Cavity: A Multi-institutional Clinicopathologic and Immunophenotypic Characterization of Five Cases and Comprehensive Literature Review

**DOI:** 10.1007/s12105-026-01931-1

**Published:** 2026-05-29

**Authors:** Prokopios P. Argyris, Ashlie E. Rubrecht, Elizabeth A. Bilodeau, Robert D. Foss, Ioannis G. Koutlas

**Affiliations:** 1 Department of Pathology, The University of Chicago Medicine, 5841 S. Maryland Avenue, MC 6040, Chicago, IL 60637 USA; 2 Department of Pathology and Laboratory Medicine, Nationwide Children’s Hospital, 700 Children’s Dr, Columbus, OH 43205 USA; 3 The Ohio State University, Columbus, OH USA; 4 Atlanta Oral Pathology, Emory Decatur Hospital, 2701 North Decatur Road, Decatur, GA 30033 USA; 5 Head and Neck Pathology, Joint Pathology Center, 606 Stephen Sitter Ave, Silver Spring, MD 20910-1290 USA; 6 Division of Oral and Maxillofacial Pathology, School of Dentistry, University of Minnesota, 16-116B Moos Tower, 515 Delaware St. SE, Minneapolis, MN 55455 USA

**Keywords:** Epithelioid sarcoma, Oral cavity sarcoma, SWI/SNF complex-deficient sarcoma, SMARCB1/INI1, Cytokeratins, Intraoral metastasis, Oral soft tissue, Jawbone sarcoma

## Abstract

**Purpose:**

Epithelioid sarcoma (ES) accounts for < 1% of all sarcomas and is characterized by predominantly epithelioid cytologic features together with epithelial immunophenotypic properties. Intraoral involvement is apparently rare with only 17 previously reported examples. Herein, the clinicopathologic and immunohistochemical features of 5 additional oral ES cases are presented with review of the literature.

**Methods:**

The electronic databases of the authors’ institutions were searched for archived, primary or metastatic, intraoral ES diagnoses. Five cases were identified. Clinicopathologic characteristics as well as information regarding treatment and outcome were collected.

**Results:**

Among the 5 cases, 3 occurred in women and 2 in men (M: F ratio = 1:1.5, age mean = 42.8 years; range = 11–79 years); 3 were primary, proximal-type, and 2 metastatic. Two primary lesions involved the tongue and 1 the buccal mandibular vestibule, whereas both metastatic ES cases affected the mandibular gingiva/alveolar mucosa. Lesion size ranged between 0.4 and 3.8 cm (mean = 1.9 cm). Microscopically, all lesions featured sheets and lobules of variably pleomorphic, epithelioid-to-spindled and occasionally rhabdoid cells with enlarged round-to-oval nuclei, vesicular or coarse chromatin, and frequent acidophilic macronucleoli, together with abundant eosinophilic cytoplasm. Mitotic activity was overall low in primary oral ES lesions but frequent in metastatic tumors; necrosis was present in 3 cases. The stroma ranged from fibrous to fibromyxoid. By immunohistochemistry, all cases showed strong and diffuse positivity for cytokeratins and EMA, together with uniform loss of nuclear SMARCB1 expression. Molecular testing in a primary lingual ES revealed somatic biallelic loss on 22q11.22-q11.23 involving the *SMARCB1* gene, together with loss of 12p. Among the 5 patients with oral ES, 1 remained disease free, 2 were alive with multifocal metastases, and 1 succumbed to the disease after a mean follow-up period of 19.7 months (range = 10–27 months). The remaining patient was lost to follow-up.

**Conclusions:**

Intraoral involvement by ES may infrequently occur either in the setting of primary or metastatic disease. The epithelioid, spindled, and, occasionally, rhabdoid cytomorphology of ES in conjunction with the aberrant immunoexpression of cytokeratins and EMA may cause a major diagnostic pitfall in oral biopsy specimens. A broad panel of epithelial and non-epithelial immunohistochemical markers, including SMARCB1, together with evaluation for surface dysplasia and a detailed clinical history, are necessary for exclusion of histologic mimics, establishing accurate diagnosis, and proper patient care.

## Introduction

Originally described by Enzinger in 1970 [1], epithelioid sarcoma (ES) is currently defined in the most recent W.H.O. classification of soft tissue and bone tumors as a malignant mesenchymal neoplasm of uncertain differentiation demonstrating partial or complete epithelioid cytologic and immunophenotypic characteristics [2]. ES comprises < 1% of all sarcomas in adults [2, 3] and 4–8% of non-rhabdomyosarcomatous soft tissue sarcomas in children and adolescents [4]. Two clinically, histologically, and biologically distinct subtypes of ES are recognized, namely the classic (conventional; distal) and proximal variants [2, 5–7]. Irrespective of subtype, ES is characterized by 22q11.23 deletions leading to inactivation of *SMARCB1* (*INI1*) gene and subsequent loss of detectable SMARCB1 nuclear expression by immunohistochemistry (IHC) [8–10]. Additionally, decreased or lost SMARCB1 nuclear expressivity may occur epigenetically due to negative regulation of SMARCB1 transcripts by miR-765 [11]. Rarely, ES with intact *SMARCB1* may harbor mutations of other SWI/SNF chromatin-remodeling complex molecules, such as *SMARCA4* (*BRG1*), *SMARCC1* (*BAF155*), or *SMARCC2* (*BAF170*) [12].

Clinically, classic ES typically involves the distal extremities, chiefly the forearm, wrist and hand, of adolescents or young adults with a predilection for men (M: F ratio = 2:1) and a median age of 30 years, although a markedly broad age range has been noted [13]. Classic ES commonly presents as indurated, solitary or multiple, slow-growing, usually painless nodules, or plaque-like lesions involving the skin and subcutis, often resulting in non-healing superficial ulceration [1, 2, 5, 14]. Classic ES may also arise deeply within the extremities or tenosynovial tissues, extending along nerves and fascial planes [13, 15]. Microscopically, classic ES comprises a nodular proliferation of large, ovoid or polygonal, epithelioid cells and plump spindle-shaped cells with central degeneration and/or geographic necrosis, imparting a pseudogranulomatous pattern reminiscent of granuloma annulare [1, 3, 13, 16]. Cytologic atypia is, overall, mild and mitotic activity low [2, 13, 16]. In contrast, proximal-type ES is less frequent, tends to affect slightly older individuals (median age = 40 years; M:F ratio = 1.6:1) and favors the pelvis, perineum, axilla, mediastinum, or genital tract, with only rare involvement of the distal extremities [6, 7, 16]. Proximal ES characteristically presents as a large, deep-seated, infiltrative soft tissue mass with hemorrhage and necrosis, and pursues a more aggressive clinical course [2, 6, 7, 13, 14, 16]. The histomorphologic characteristics of proximal-type ES encompass a multinodular or sheet-like growth of enlarged, predominantly epithelioid and often rhabdoid cells with nuclear pleomorphism and prominent nucleoli [2, 3, 6, 13, 15–18].

ES of the head and neck region is markedly uncommon accounting for < 1% of all ES cases [10, 19–21]. Sites of involvement include the scalp [22], orbit [19, 23], maxillary sinus [19, 21], parotid gland [21], oropharynx [21], parapharyngeal space [24], external auditory canal [5, 20, 25], and neck [26]. ES of the oral cavity is exceedingly rare with only sparse reports in the English literature [21, 27–35]. Herein, we present a multi-institutional clinicopathologic analysis of 5 cases of ES with oral involvement, together with a comprehensive review of the pertinent literature

## Materials and Methods

### Case Identification and Selection

The electronic databases of five institutions, including four oral/head and neck pathology laboratories and one specialized pediatric hospital, were searched for archived, formalin-fixed paraffin-embedded cases of primary or metastatic ES involving the oral cavity. Inclusion criteria comprised (1) availability of original hematoxylin and eosin (H&E) and IHC-stained histologic slides and/or tissue blocks, (2) a neoplastic cell population with epithelioid, spindled and/or rhabdoid cytomorphology, (3) convincing tumor immunoreactivity for at least one of the following epithelial differentiation markers: cytokeratin AE1/AE3, MNF116, OSCAR, CAM5.2, CK5/6, or epithelial membrane antigen (EMA), and (4) loss of SMARCB1 (INI1) expression by IHC. By definition, examples of oral spindle-cell (sarcomatoid) squamous cell carcinoma (SCC) were excluded from this study [36]. Five cases fulfilling the above criteria were identified and information regarding patient demographics, anatomic site, size and clinical presentation, available panel of IHC markers, as well as treatment and follow-up was collected (Table 1).

**Table 1:** Collective presentation of the demographic, clinicopathologic and immunophenotypic characteristics, together with treatment and available follow-up information of previously reported cases of intraoral ES (N = 17), including the five examples of the current study

Author/ # of cases	Age (years)/ Gender	Clinical Presentation	Location	Size (cm)	Histopathologic Diagnosis	IHC Findings	Treatment	Follow-up/Outcome
Jameson et al. [27] n = 1	20/M	Painless chronic ulcer present for 1 year; periapical radiolucency associated with vital maxillary central incisors	Anterior hard palate	1.0	ES, primary	CAM5.2( +), vimentin( +)	Wide surgical excision	Two local recurrences at 12 and 48 months; 60 months, NED
Leroy et al. [28] n = 1	35/F	Painful, non-ulcerated, nodular lesion of several months duration	Tongue	2.5	ES, primary	Cytokeratin (KL1 +), EMA( +), vimentin( +)	Hemiglossectomy, brachytherapy	Cervical LN METs at 36 months; 84 months, DOD with distant METs (scalp, lung, brain)
Winter et al. [29] n = 1	64/M	Painful, firm nodule; dysphagia	Tongue	NA	ES, metastatic; hand primary 24 months prior	Cytokeratin( +), EMA( +), actin( +), vimentin( +)	Palliative surgical resection	8 months, DOD with pleural METs
Ozdemir et al. [30] n = 1	45/M	Painful, firm, nodulo-ulcerative lesion of 4-month duration; dysphagia	Tongue	NA	ES, metastatic; arm primary	Cytokeratin( +), CD34( +), vimentin( +)	Surgical excision, chemotherapy	NA, AWD with distant METs (scalp, abdominal wall, lung)
Kao et al. [31] n = 1	57/M	Rapidly growing, painful, gingival swelling of 4-month duration; minimal alveolar bone destruction	Mandibular gingiva; right molar region	2.0	ES, metastatic; wrist primary with axillary LN METs 18 months prior	AE1/AE3( +), EMA( +), vimentin( +)	Surgical excision, chemotherapy	24 months, NED
Sukpanichyingyong et al. [32] n = 1	31/F	Multifocal, painless, focally ulcerated, erythematous, gingival nodules; tooth mobility	Maxillary buccal/palatal gingiva; right central incisor to canine and molar region	NA	ES, metastatic; forearm primary 24 months prior	AE1/AE3( +), EMA( +), CD34( +)	Palliative care	6 months, DOD
Kazi et al. [33] n = 1	17/M	Non-healing, nodulo-ulcerative lesion of 1-month duration	Tongue	2.5	ES, primary; proximal type	AE1/AE3( +), CD34( +), ERG( +) INI1 loss	Partial glossectomy, bilateral selective neck dissection, adjuvant radiation	6 months, NED
Brar et al. [34] n = 1	23/F	Slow-growing, painful, indurated, ulcerated mass of 3-month duration	Tongue	1.5	ES, proximal type; primary	AE1/AE3( +), CAM5.2( +), CK5/6( +), p16( +), ERG( +), CD99( +), INI1 loss	Partial glossectomy, bilateral selective neck dissection, adjuvant radiation	18 months, NED
Shi et al. [21]* n = 8	6 M:2F Mean age = 24.8 (9–47)	Exophytic, pedunculated, firm, ulcerated mass; ipsilateral submandibular lymphadenopathy and scalp METs Rapidly growing mass following prior local excision	Tongue (n = 3) Buccal mucosa (n = 2) Gingiva (n = 1) FOM (n = 1) Lip (n = 1)	Mean size = 3.2 (1.5–6.2)	ES, classic type (n = 6); 4 primary; 2 metastatic from foot and shoulder primary ES, proximal type (n = 2); primary	AE1/AE3(+ ; 8/8), FLI1(+ ; 8/8), CD34(+ ; 7/8), vimentin(+ ; 8/8), INI1 loss (8/8), S100(+ ; 3/8), Ki67 = 10–70%; mean = 27.5%	Wide surgical excision, ± LN dissection, ± adjuvant radiation	Not specified
Ray et al. [35] n = 1	55/F	Diffuse, painless, expansive, firm, focally ulcerated growth of 2-year duration; facial asymmetry	Mandibular alveolar mucosa	NA	ES, proximal type; primary	EMA( +), CD34( +), BRG1 and H3K27me3 retained, INI1 loss	NA	NA
*Current study (Argyris *et al*.); n* = *5*								
Case 1	17/F	Firm submucosal swelling	Tongue	3.8	ES, proximal type; primary	AE1/AE3( +), CD34(focal +), ERG(focal +), S100(focal +), INI1 loss	Chemotherapy only; patient refused glossectomy	27 months, AWD with lung METs, under chemotherapy
Case 2	11/M	Destructive lytic, expansile lesion of left anterior mandible	Mandibular gingiva and alveolar bone	1.5	ES, proximal type; metastatic from retroperitoneal primary	AE1/AE3( +), CK8/18( +), CD34( +), Glut-1( +), EMA( +), INI1 loss, Ki67 = 30–40%	Palliative care	10 months, DOD with CNS METs
Case 3	39/M	Poorly-demarcated, hemorrhagic, violaceous, nodular lesions	Mandibular gingiva and alveolar mucosa	2.6	ES, metastatic; arm primary	OSCAR( +), ERG( +), INI1 deficiency	Surgery with adjuvant chemoradiation	19 months, AWD with cutaneous, lung, and skeletal bone METs
Case 4	68/F	Asymptomatic, solitary, well-demarcated, circular macule of 4-month duration	Tongue	0.4	ES, proximal type; primary	AE1/AE3( +), CAM5.2( +), CA-125( +), ERG( +), WT-1(focal +), p53(focal +), INI1 loss, Ki67 < 5%	Partial glossectomy	23 months, NED
Case 5	79/F	Submucosal mass in contact with mental foramen and nerve	Left buccal mandibular vestibule, premolar region	1.5	ES, proximal type; primary	AE1/AE3( +), EMA( +),CD34( +), ERG( +), vimentin( +), FOSB(focal +), INI1 loss	Patient refused treatment	Lost to follow-up

*In the current study, detailed information regarding the clinical presentation was provided for only 2 of 8 intraoral ES cases. All patients with head and neck ES underwent wide surgical excision, 9 of 12 also received lymph node dissection, 7 of 12 adjuvant radiation therapy, and 3 of 12 neoadjuvant chemotherapy

ES, epithelioid sarcoma; MET, metastasis; FOM, floor of mouth; CNS, central nervous system; NA, not available; NED, no evidence of disease; DOD, dead of disease; AWD, alive with disease

## Literature Search

Publicly available electronic databases, including PubMed, Medline and Google Scholar, were searched during the period 01/1990-01/2026 for previously reported cases of oral ES using the following combination of keywords: “epithelioid sarcoma” and “oral cavity”, “oral mucosa”, “intraoral”, “tongue”, “gingiva”, “palate”, “vestibule”, or “mandible/maxilla”. Inclusion criteria for the current review encompassed case reports and series published in the English literature with adequate documentation of the clinical, histopathologic and immunophenotypic features of the lesion(s). Oral ES lesions reported together with extraoral cases and published abstracts lacking detailed description of the demographic and clinical findings were excluded [4, 37]. Additionally, a case of presumed proximal-type ES involving the posterior mandibular gingiva of an individual with prolonged history of tobacco smoking and areca nut chewing, without SMARCB1 IHC status, probably represents sarcomatoid SCC, and therefore, excluded [38]. Ten publications fulfilling the above criteria were identified, including 9 single case reports [27–33, 35, 39] and 1 case series [21], leading to a total of 17 previously reported examples of oral ES. These cases are summarized in Table 1.

## Results

### Multi-institutional Cohort

#### Demographic and Clinical Findings

**Fig. 1:**
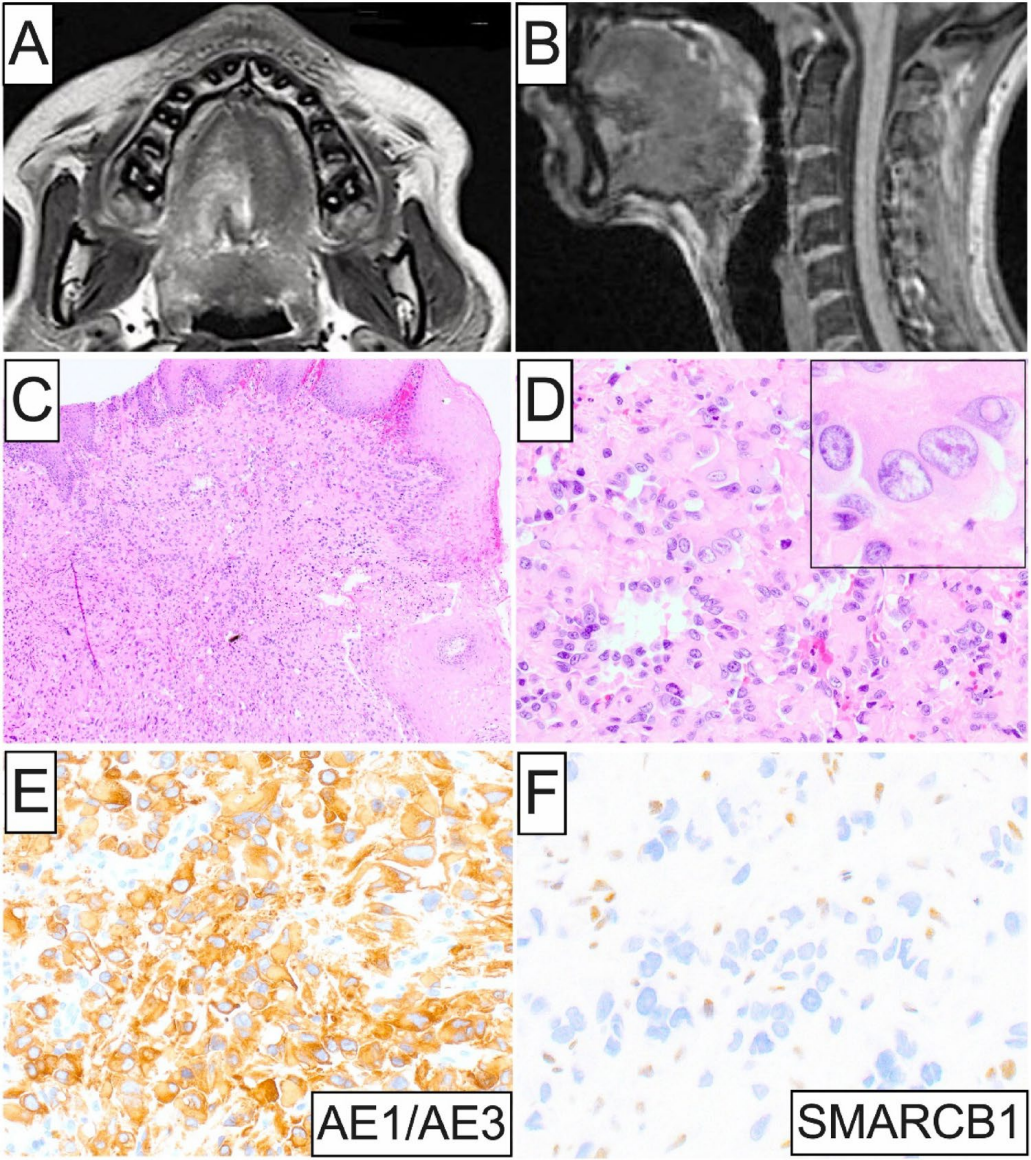
Imaging, histopathologic and IHC findings of Case 1. (**A**) and (**B**) Axial and sagittal MRI views showing a 3.8 cm lingual submucosal mass; (**C**) and (**D**) Low- and high-power photomicrographs displaying a focally ulcerated poorly-defined lesion composed of sheets of predominantly epithelioid cells with enlarged pleomorphic nuclei (inset), vesicular to coarse chromatin, prominent nucleoli, and abundant eosinophilic cytoplasm; (**E**) and (**F**) Strong and diffuse AE1/AE3 immunostaining together with loss of SMARCB1 nuclear expression in sarcoma cells. Retained SMARCB1 expression is observed in background endothelial and inflammatory cells

**Fig. 2:**
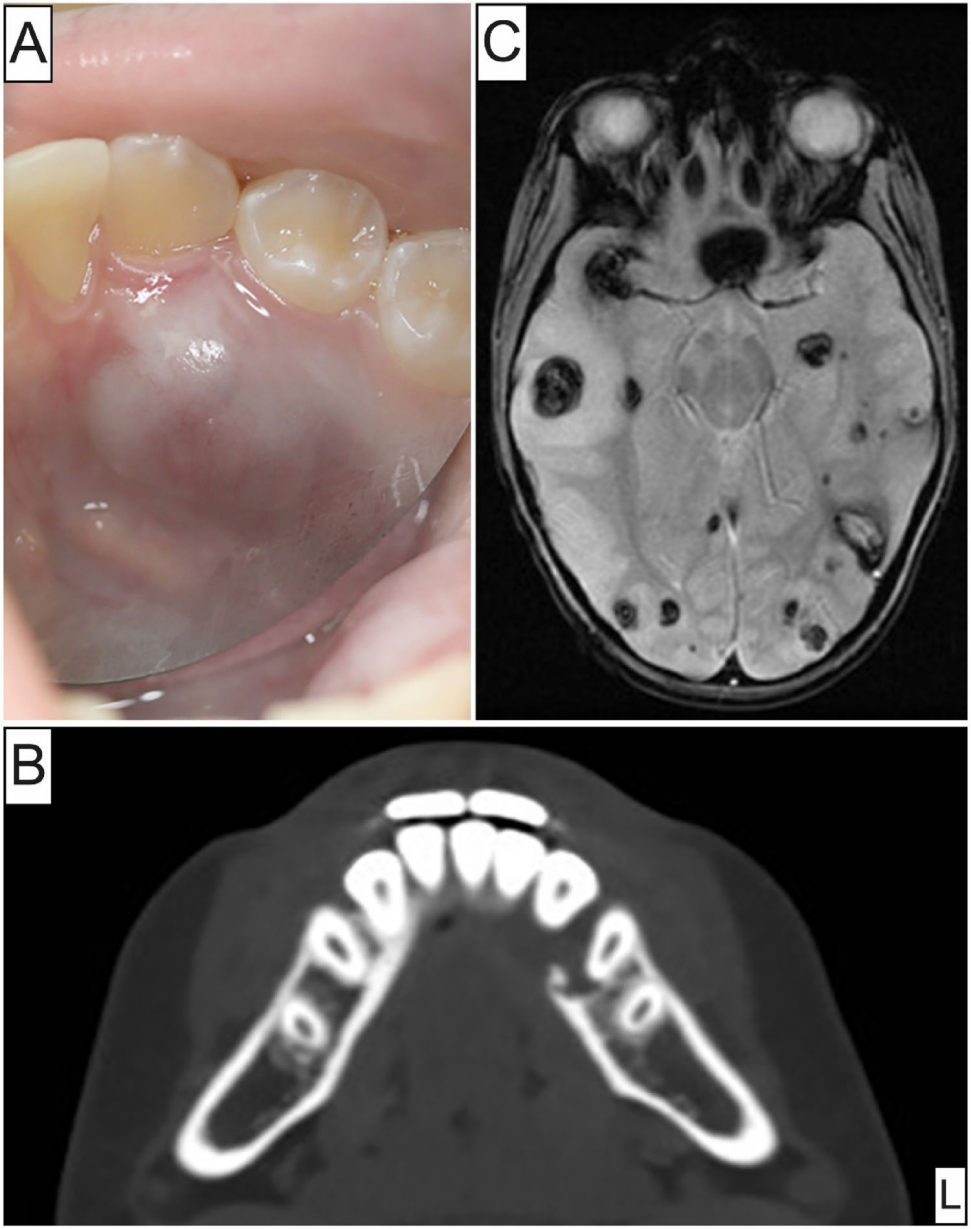
Clinico-radiologic presentation of Case 2. (**A**) Metastatic ES from retroperitoneal primary presenting as localized swelling of the lingual mandibular gingiva and alveolar bone; (**B**) Axial CT view revealing an expansile, destructive, osteolytic mass involving the left body of the mandible; (**C**) Axial MRI view of the brain confirming numerous ES metastases

Among the 5 intraoral ES cases identified, 3 occurred in women and 2 in men (M: F ratio = 1:1.5) with an age mean of 42.8 years and a broad age range (11–79 years). Three oral ES were primary, proximal-type, while 2 represented oral metastases from the retroperitoneum and arm, respectively. Size ranged between 0.4 and 3.8 cm (mean = 1.9 cm). Among the 3 primary ES cases, 2 involved the tongue presenting either as firm submucosal swelling causing lingual enlargement (Fig. 1A and B) or an asymptomatic, solitary, well-demarcated, circular macule; the third presented as a submucosal mass of the buccal mandibular vestibule in association with the mental foramen and nerve. There was no evidence of associated cervical lymphadenopathy. Both metastatic ES cases affected the mandibular gingiva/alveolar mucosa. One presented as an expansile, destructive, osteolytic mass (Fig. 2A and B), while the other as poorly-demarcated, hemorrhagic, violaceous, multinodular gingival lesion (Fig. 4A). The clinico-demographic characteristics of the 5 cases of oral ES included in this cohort are summarized in Table 1.

### Histopathologic, Immunophenotypic and Molecular Characteristics

**Fig. 3:**
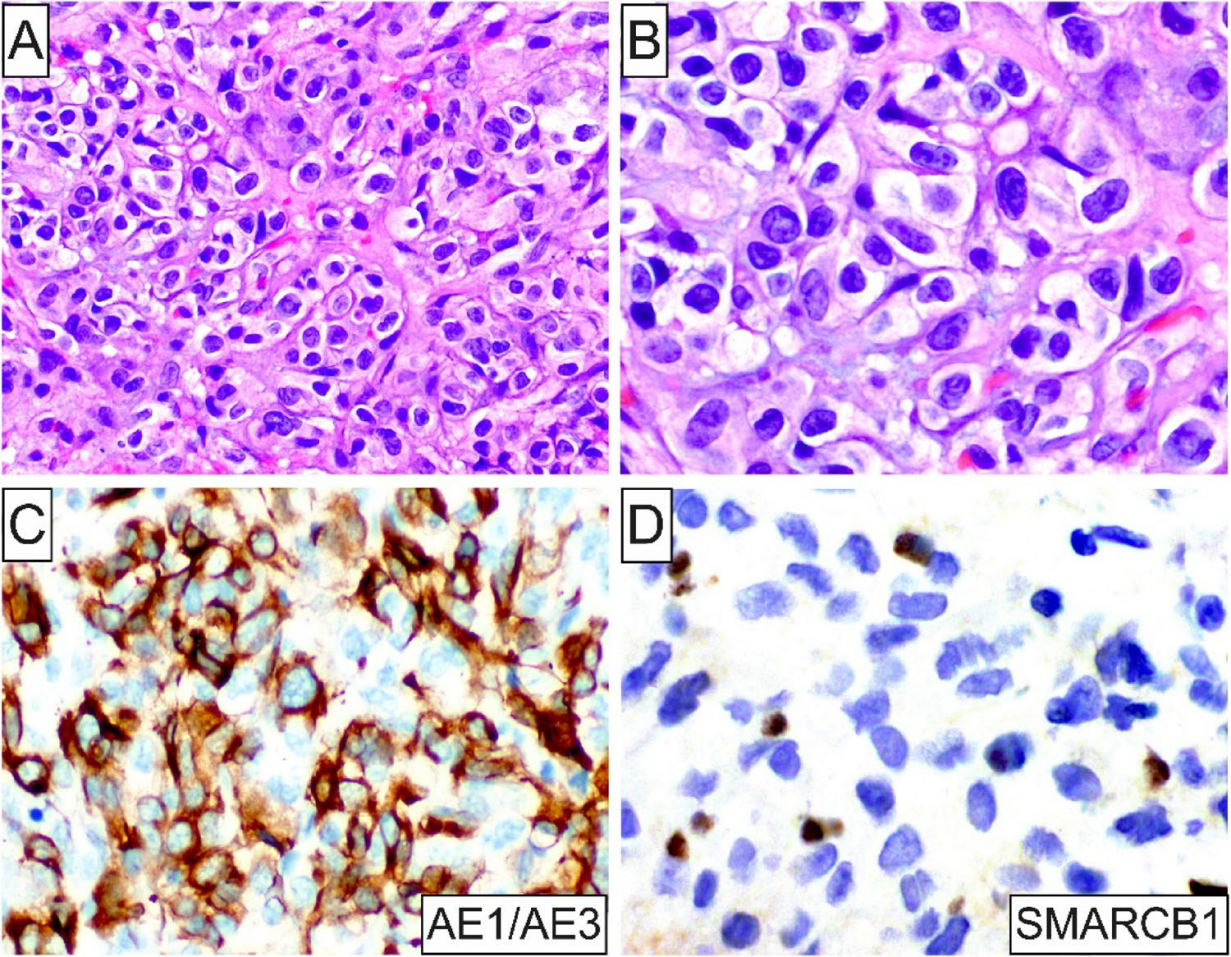
Histopathologic and IHC characteristics of Case 2. (**A**) and (**B**) Medium- and high-power photomicrographs showing a sheet-like growth of atypical epithelioid and spindled cells with hyperchromatic nuclei, occasionally visible nucleoli, and pale eosinophilic to optically-clear cytoplasm, supported by well-vascularized fibrous stroma; (**C**) and (**D**) Strong and diffuse AE1/AE3 immunoreactivity together with loss of SMARCB1 expression in ES cells

**Fig. 4:**
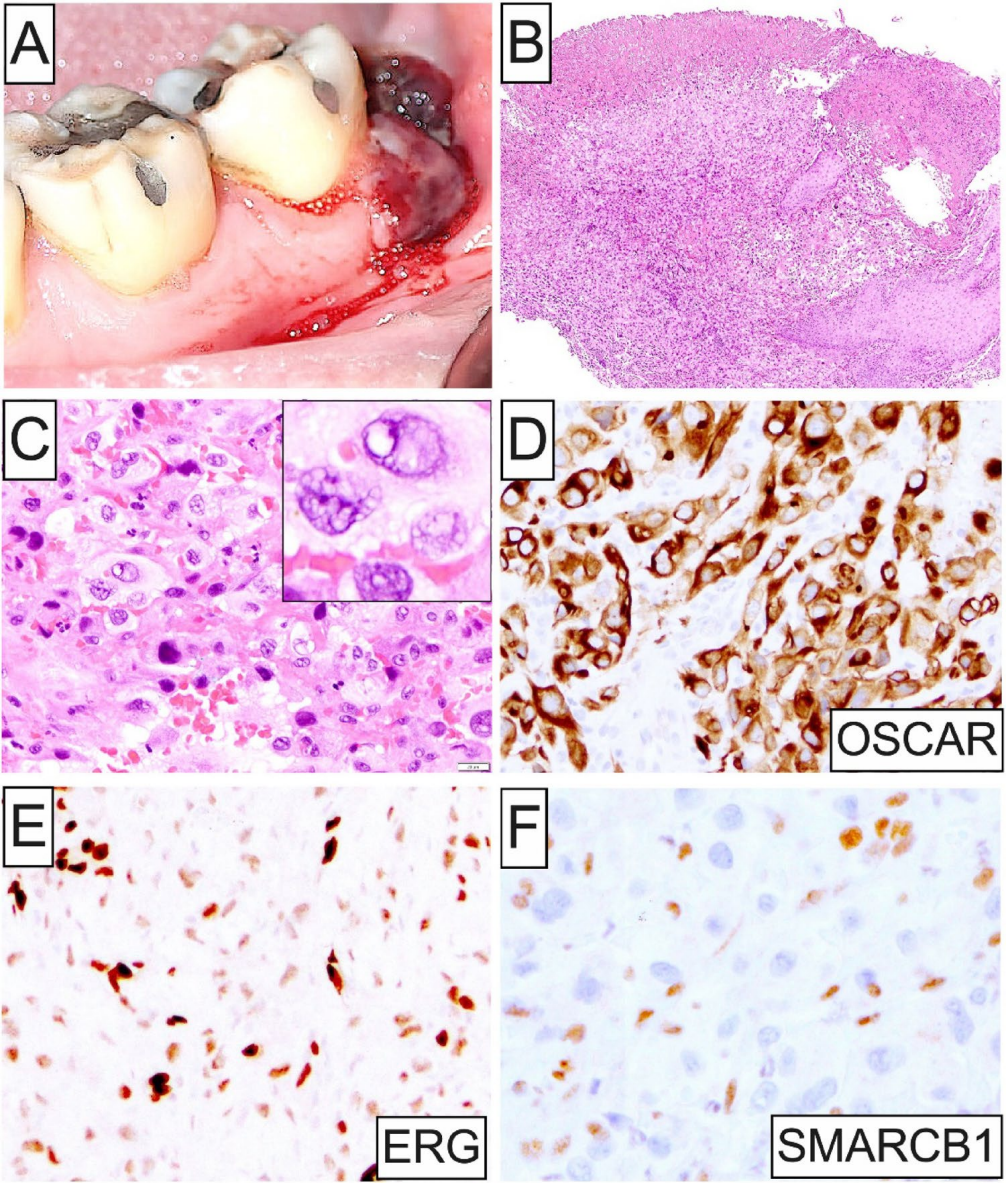
Clinicopathologic and IHC characteristics of Case 3. (**A**) Metastatic ES presenting as poorly-demarcated, hemorrhagic, violaceous, multinodular growth of the left posterior mandibular gingiva/ alveolar mucosa; (**B**) and (**C**) Low- and high-power photomicrographs depicting an ulcerated oral mucosal lesion comprising sheets of markedly pleomorphic, epithelioid and spindled neoplastic cells featuring enlarged, round-to-oval nuclei, vesicular or dense chromatin (**inset**), occasional macronucleoli, and rich eosinophilic cytoplasm. Necrosis and zones of hemorrhage are present; (**D**-**F**) ES cells demonstrate aberrant OSCAR cytokeratin expression in conjunction with ERG (clone EPR3864) immunoreactivity and loss of SMARCB1

**Fig. 5:**
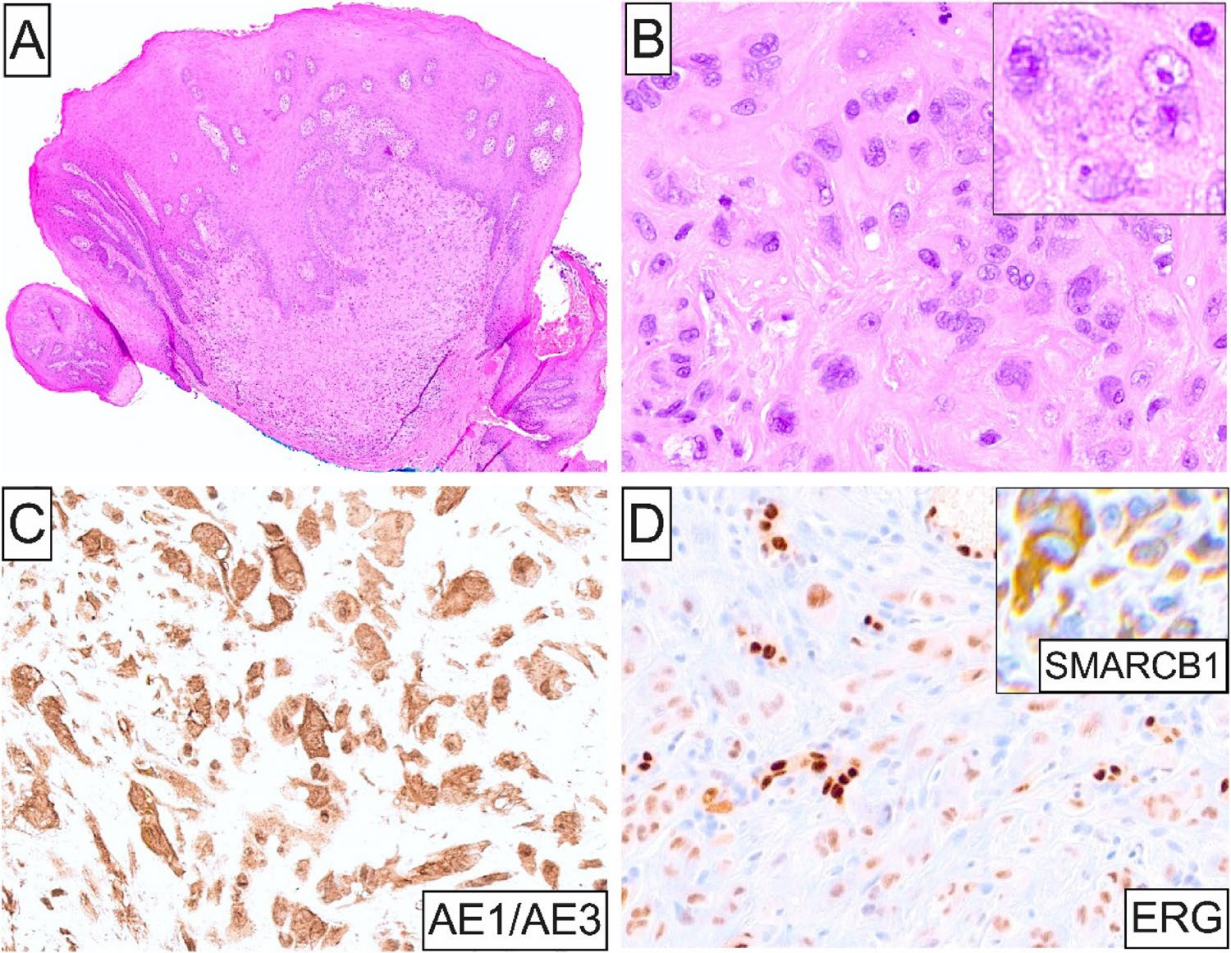
Histopathologic and IHC characteristics of Case 4. (**A**) and (**B**) Primary ES presenting as a 0.4 cm circular macule of the dorsal tongue comprising large epithelioid and, in areas, rhabdoid neoplastic cells with pleomorphic vesicular nuclei, 1–2 acidophilic macronucleoli, and densely eosinophilic cytoplasm; (**C**) and (**D**) Lesional cells show strong immunoreactivity for AE1/AE3, weak-to-moderate staining for ERG (clone EP111), and SMARCB1 inactivation (**inset**)

**Fig. 6:**
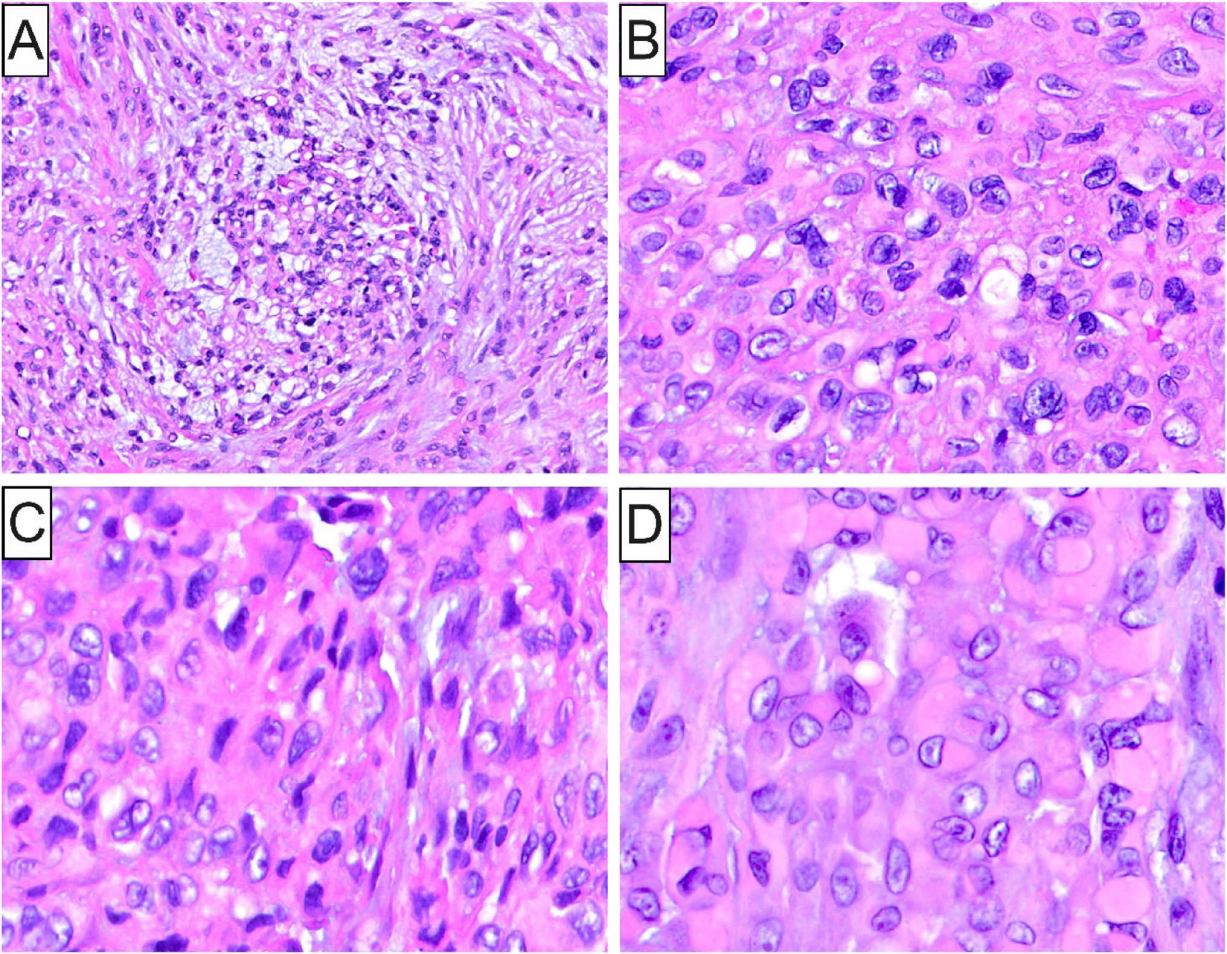
Histopathologic characteristics of Case 5. (**A**) Low-power photomicrograph depicting a vaguely lobulated “granuloma-like” cell proliferation immersed in a fibromyxoid stroma; (**B**-**D**) High-power photomicrographs highlighting the cytomorphologic characteristics of this ES composed of epithelioid, spindled and rhabdoid cells with moderately-pleomorphic nuclei, open chromatin and occasionally visible nucleoli, together with ample, densely eosinophilic (glassy) cytoplasm

Microscopically, all 5 tumors were ill-defined (Figs. 1C, 4C, 5A and 6A) and featured sheets and lobules of large, variably pleomorphic, epithelioid-to-spindled and occasionally rhabdoid neoplastic cells (Figs. 1D, 3A, 5B and 6B). Lesional cells exhibited enlarged, round-to-oval nuclei with irregular nuclear contours, vesicular or coarse chromatin, frequent acidophilic macronucleoli, together with abundant, lightly-to-densely eosinophilic cytoplasm and distinct cell membrane borders (Fig. 1D and inset, 4 C and inset, 5B and inset, 6B-D). One case of metastatic ES (case 2; Table 1) was composed of an admixed population of atypical epithelioid and spindled cells with predominantly hyperchromatic nuclei, occasionally visible nucleoli, and pale eosinophilic to optically-clear cytoplasm (Fig. 3B). Nuclear atypia and pleomorphism ranged from moderate to prominent, and mitotic activity was overall low in primary oral ES lesions but conspicuous in metastatic tumors. The surrounding stroma comprised fibrous and, in one case (case 5), fibromyxoid tissue (Fig. 6A). Three cases demonstrated areas of necrosis and two surface ulceration.

By IHC, all oral ES lesions revealed strong and diffuse positivity for cytokeratins, including AE1/AE3 (4 of 4; Figs. 1E, 3C and 5C), OSCAR (1 of 1; Fig. 4D), CAM5.2 (1 of 1), and CK8/18 (1 of 1), EMA (2 of 2), together with at least focal expression of ERG (4 of 4; Figs. 4E and 5D) and CD34 (3 of 3). Additionally, selective ES cases were immunoreactive for Glut-1, CA-125, WT-1 (focal), p53 (focal; wild-type pattern), S100 (focal), and FOSB (focal). Proliferative index Ki67 was < 5% and 30–40% in 1 primary (case 4) and 1 metastatic oral cavity ES (case 2), respectively. Notably, abnormal loss of SMARCB1 (INI1) nuclear expression characterized all 5 oral ES cases (Figs. 1F, 3D, 4F and 5D inset; Table 1). The panel of negative IHC markers included desmin (4 of 4), myogenin (2 of 2), myoD1 (1 of 1), p63 (4 of 4), SMA (4 of 4), CD31 (3 of 3), S100 (3 of 3), ALK (1 of 1), SALL4 (1 of 1), and CAMTA1 (1 of 1) among others.

Molecular testing (Somatic Disease/Germline Comparator) was performed in case 1, a primary ES of the tongue, and revealed somatic interstitial biallelic loss on cytogenetic locus 22q11.22-q11.23 involving the *SMARCB1* gene, together with loss of 12p.

## Treatment and Follow-up

Among the 3 patients with primary ES of the oral cavity, one was treated with partial glossectomy and remains disease free 23 months post-surgery. The adolescent female with lingual ES received only chemotherapy, developed bilateral pulmonary metastases ~ 4–5 months post-diagnosis for which she underwent resection, and is currently alive with disease (AWD) after 27 months of follow-up. The third patient refused treatment and has been lost to follow-up.

Regarding the two individuals with oral metastatic ES, the 11-year-old boy with retroperitoneal ES metastatic to the gingiva and alveolar bone succumbed 10 months post-diagnosis owing to multiple brain metastases (Fig. 2C), while the 39-year-old man with arm primary and gingival metastasis, received surgery with adjuvant chemoradiation and was AWD 19 months post-diagnosis with multifocal cutaneous, pulmonary, and skeletal bone lesions (Table 1).

## Literature Review Analysis on Oral ES

Seventeen (*N* = 17) reported examples of oral cavity ES were identified fulfilling the inclusion criteria of this study [21, 27–35]; 11 (64.7%) occurred in men and 6 (35.3%) in women (M: F ratio = 1.8:1) with a mean age of 37.2 years (age range = 9–64 years; Table 1). A site predilection for the tongue was noted (8 of 17; 47%), followed by the gingiva (3 of 17; 17.6%), buccal mucosa (2 of 17; 11.8%), and one each (5.9%) for the palate, floor of mouth, lip and alveolar mucosa. The majority of cases (11 of 17; 64.7%) were primary, while 6 (35.3%) cases comprised intraoral metastases from ES originating mainly from the upper distal extremities (4 of 6) [29–32], and less frequently the shoulder or foot (1 case each) [21]. The clinical characteristics of oral cavity ES varied with most cases presenting as a painful and, less commonly painless, firm submucosal swelling, indurated chronic ulcer, or nodulo-ulcerative lesion ranging in duration from 1 to 24 months [33, 35], and measuring on average 2.1 cm (range = 1.0–6.2 cm). Dysphagia was reported in two cases of submucosal lingual ES [29, 30], while another case involving the mandibular alveolar mucosa caused facial asymmetry [35]. Notably, all 3 previous examples of gingival ES comprised metastatic lesions and manifested as solitary or multifocal, firm, sessile or pedunculated, erythematous nodules or masses with surface ulceration [21, 31, 32]; associated tooth mobility was reported in 1 case [32]. Gingival metastases developed 18–24 months following primary diagnosis [31, 32].

Available IHC data for the 17 previously published cases of oral ES are also summarized in Table 1. All intraoral ES tumors were invariably positive for epithelial markers including pancytokeratin (15 of 15), e.g., AE1/AE3, EMA (5 of 5), CAM5.2 (2 of 2), and CK5/6 (1 of 1), as well as CD34 (11 of 12), FLI1 (8 of 8), and ERG (2 of 2). Immunoreactivity for S100 (3 of 8), actin (1 of 1), p16 (1 of 1), and CD99 (1 of 1) was reported in selective cases. Ki67 was assessed in 8 cases and ranged between 10 and 70% (mean = 27.5%) [21]. Loss of nuclear INI1 was confirmed in all 11 cases tested [21, 33–35], whereas expression of BRG1 and H3K27me3 was retained (1 of 1) [35].

Detailed treatment information was available in only 8 of 17 oral ES cases. Three patients received partial or hemiglossectomy and adjuvant radiation therapy [28, 33, 34], with 2 of them also receiving bilateral selective neck dissection, 2 were treated with surgical excision and adjuvant chemotherapy [30, 31], while 1 patient underwent only surgery [27]. Two individuals with metastatic ES of the tongue and gingiva, respectively, received palliative treatment [29, 32]. In the case series by Shi et al. [21], all 8 oral cavity ES were treated with at least wide surgical excision but treatment modalities for each of the cases were not further specified. Locoregional recurrences were reported in one primary ES affecting the hard palate [27], while disseminated metastases to the scalp, lung, abdominal wall, pleura or brain were observed in association with 3 lingual ES cases of which 2 were metastatic [29, 30] and 1 primary [28]. Collectively, 4 of 8 patients with oral ES were free of disease, 3 were dead of disease (DOD), and 1 AWD after a mean follow-up period of 29.4 months (range = 6–84 months) [27–34].

## Discussion

ES is a rare mesenchymal malignancy with an estimated annual incidence rate of 0.02–0.05 cases per 100,000 in Europe and USA [40]. It accounts for roughly 0.4% of all sarcomas involving the head and neck region [21] and merely 0.9% of those occurring in the oral cavity [41]. Review of the English literature disclosed only 17 previously reported examples of oral cavity ES. Comparison of the epidemiologic and clinical findings of our series to the previously documented examples reveals overt similarities regarding patient age and site distribution (data summarized in Table 1). Specifically, in both series, ES of the oral cavity showed a preponderance for young adults with a mean age approximating 40 years (42.8 and 37.2 years, respectively), and a markedly broad range spanning from the 1st -2nd decades of life to the 7th and 8th decades. Approximately 27% (6 of 22) of oral ES cases occurred in children and adolescents. Irrespective of patient age and akin to certain other sarcoma subtypes such as alveolar soft part sarcoma [42–45] and *GLI1*-altered soft tissue tumor [46–48], primary ES of the oral cavity exhibits a strong predilection for the tongue which accounts for 45.5% (5 of 11) [21, 28, 33, 34] to 66.7% (2 of 3, current study) of all primary tumors. As anticipated, the striking majority of metastatic ES to the oral cavity affected the gingival/alveolar mucosa (5 of 8; 62.5%, collective data), followed by the tongue (3 of 8; 37.5%) [21, 29, 30]. Overall, sarcomas metastasizing to the oral soft tissues and/or jawbones are considered exceedingly rare encompassing only 3.9% of oral cavity sarcomas [41] and merely 0.7% of all intraoral metastatic malignancies [49]. Finally, review of the literature revealed a male preponderance of oral ES cases (M: F ratio = 1.8:1) which is in keeping with ES of extraoral sites [13, 18]. The slight female predilection (M: F ratio = 1:1.5) observed in the present series is most likely attributable to limited sample size.

Genomic inactivation with subsequent loss of nuclear expression of SMARCB1 (INI1), a core subunit of the SWI/SNF chromatin remodeling complex, characterizes over 95% of ES [9, 10, 17], as seen in all 5 cases of the current cohort. One should take into consideration that confirmatory INI1 immunostain was available only in 11 of 17 previously reported oral ES cases [21, 33–35], whereas diagnosis in the remaining lesions was established solely on histomorphology in conjunction with other, less specific, immunophenotypic findings, e.g., AE1/AE3, CAM5.2, EMA, and/or CD34 expression [27–32].

Loss of INI1 expression is not pathognomonic for ES and has been documented in a broad variety of epithelial and mesenchymal malignancies with diverse histopathologic characteristics and biologic properties [10, 50, 51]. For instance, virtually all malignant rhabdoid tumors (MRTs) [10, 52] and poorly-differentiated chordomas [53], together with a subset of epithelioid malignant peripheral nerve sheath tumors [50, 51, 54, 55], myoepithelial carcinomas [9] and extraskeletal myxoid chondrosarcomas with prominent rhabdoid features [56] harbor *SMARCB1* inactivation demonstrable by IHC. Furthermore, SMARCB1-deficiency has been documented in a skull-based chondrosarcoma representing somatic-type malignancy arising from metastatic testicular seminoma [57]. Among the aforementioned SMARCB1-deficient mesenchymal neoplasms, special emphasis will be given to MRT owing to considerable histomorphologic and immunophenotypic overlap with ES.

MRTs typically arise in the kidney, central nervous system (atypical teratoid/ rhabdoid tumor), and deep soft tissues, and mainly affect infants and young children with the majority of cases diagnosed by the age of 3 years [50, 52, 58]. Extrarenal and extracranial MRTs involving the head and neck are rare and usually present as rapidly-growing friable masses with a predilection for the oral cavity and neck [10, 59, 60]. Histologically, MRTs are characterized by an unencapsulated infiltrative population of predominantly rhabdoid cells featuring abundant, glassy, eosinophilic, inclusion-like cytoplasm, eccentric vesicular nuclei with prominent nucleoli, and brisk mitotic activity [10, 58, 60, 61]. Large epithelioid and/or spindled cytomorphology can also be seen, while some MRTs may be dominated by primitive undifferentiated round cells (small blue cells) with only sparse rhabdoid cells [10, 61]. Lesional cells display a dyscohesive sheet-like, trabecular, or occasionally alveolar growth pattern with frequent necrosis and vascular infiltration [58, 60]. Similar to ES, MRTs aberrantly express cytokeratins and EMA, although keratin immunoreactivity is more common with CK8/18 [13, 58, 60, 62]. Distinction between proximal-type ES and MRT is based on a combination of clinical, histologic, immunophenotypic and molecular findings. As also shown in our series, ES occurs in adolescents and young to middle-aged adults, tends to show multinodularity, and chiefly comprises large epithelioid cells with a limited rhabdoid component [13, 16, 21]. Of note, MRT demonstrates a rapidly progressive clinical course with widespread dissemination and dismal outcomes; 5-year survival rate is only 15–20% [10, 58, 62]. By IHC, ERG expression is identified in 40–50% of ES cases [63, 64] but is typically absent in MRT, while SALL4 immunoreactivity is observed in most MRTs (71%) but is rare in ES [65]. Finally, although both tumors feature loss of INI1 expression by IHC, *SMARCB1* inactivation in ES is mostly driven by monoallelic or biallelic deletions at the 22q11.23 locus, as shown in case 1, together with epigenetic silencing by miR-765 [2, 11]. In contrast, MRTs invariably exhibit homozygous *SMARCB1* deletions, translocations, or other aberrations, and frequent *TP53* mutations [58, 60]. Furthermore, ~ 30% of MRTs harbor germline *SMARCB1* mutations in association with rhabdoid tumor predisposition syndrome type 1 (RTPST1) [66, 67].

The main differential diagnostic consideration for ES of the oral cavity is the spindle-cell (sarcomatoid) variant of SCC. Indeed, strong and diffuse immunoreactivity for epithelial differentiation markers, i.e., AE1/AE3, OSCAR, CAM5.2, CK5/6, and EMA, in oral ES in conjunction with the epithelioid and spindled cytomorphology may pose a major diagnostic pitfall, particularly in extensively ulcerated lesions and/or small biopsy specimens. Moreover, distinction between oral ES and sarcomatoid SCC can be confounded by the absence of a concomitant, well-differentiated SCC component and epithelial dysplasia in 50% of oral sarcomatoid SCCs [36]. Immunoreactivity for p63 or p40 can be proven diagnostically useful, although it is only identified in 31.6% and 57.9% of oral cavity sarcomatoid SCCs, respectively [68–71]. Evidently, in contrast to oral ES, INI1 nuclear expression is retained in spindle-cell SCC. Notably, most ES cases involving the oral cavity occur in adolescents and young adults (Table 1), whereas oral SCC would be uncommon in the 4th decade and exceptionally rare during the first 3 decades of life in individuals lacking an inherited predisposing condition, e.g., Li–Fraumeni syndrome, Fanconi anemia, dyskeratosis congenita, and xeroderma pigmentosum [72]. Finally, primary or metastatic ES should be distinguished from SWI/SNF complex-deficient pulmonary undifferentiated carcinoma metastasizing to the oral cavity, especially the gingiva [73].

Special emphasis should be given in discerning oral ES from rhabdomyosarcoma with *TFCP2* rearrangement, a recently described, distinct and clinically aggressive sarcoma variant characterized most commonly by a biphasic epithelioid and spindle cell morphology, extensive bone and soft tissue destruction, and a strong predilection for young adults (median age: 25 years, range: 11–86 years) [46, 74–81]. *TFCP2*-rearranged rhabdomyosarcomas harbor fusions of *TFCP2* at the 5ʹ end to *FUS* or *EWSR1* and virtually all cases are diffusely positive for pancytokeratins, e.g., AE1/AE3, OSCAR, MNF116, and desmin, as well as myogenin and/or MYOD1, with MYOD1 demonstrating higher sensitivity. Occasionally, ALK mRNA and protein overexpression has been reported [46, 74–81]. In contrast to ES, SMARCB1 expression is retained in *TFCP2*-rearranged rhabdomyosarcomas [79, 80], which in conjunction with rhabdomyoblastic markers can be diagnostically useful.

Other entities necessitating special mention and consideration in the differential diagnosis of oral ES, particularly in older individuals, include mucosal melanoma and epithelioid angiosarcoma. Melanoma of either mucosal origin or metastatic may feature all three cell phenotypes encountered in ES, i.e., epithelioid, spindle, and rhabdoid, while melanomas with epithelioid/rhabdoid features may also show focal-to-diffuse keratin expression akin to ES [82, 83]. However, unlike ES, melanomas typically exhibit strong and diffuse immunoreactivity for S100 and SOX10, often in addition to HMB45, melan-A, and PRAME [84, 85]. Furthermore, BRAF VE1 IHC can be helpful in the context of metastatic cutaneous melanoma to the oral cavity, while detection of underlying *BRAF*, *NRAS*, *NF1*, and UV signature mutations, i.e., C-to-T transitions at dipyrimidine sites, can be crucial in the setting of metastatic undifferentiated/ dedifferentiated melanoma [86–89]. Epithelioid angiosarcomas can also demonstrate strong cytokeratin immunostaining in up to 50% of cases and they are invariably positive for pan-endothelial IHC markers CD31, CD34, and ERG [90–93]. Despite its frequent positivity for CD34 and ERG, as also observed in our cases, ES is usually CD31 negative [63, 64]. INI1 nuclear expression is retained in both mucosal melanomas and epithelioid angiosarcomas.

Wide surgical excision with clear margins remains standard of care for ES with reported 5- and 10-year overall survival rates of 45–70% and 45–66%, respectively [2, 94–96]. Of note, about 22–30% of cases present with regional or distant metastases at diagnosis [95, 96]. Multimodal therapeutic regimens, including adjuvant radiation therapy alone or in combination with anthracycline-based chemotherapy, i.e., doxorubicin and ifosfamide, are commonly utilized in advanced stage disease. Reported negative prognosticators for extraoral ES comprise older patient age, tumor size greater than 5 cm, high-grade features, i.e., high mitotic count and necrosis, proximal-type histomorphology, and lymph node involvement [2, 8, 97, 98]. The clinical efficacy of α-PD-L1/PD-1 immunotherapy in ES is for the most part limited with only rare exceptions demonstrating marked response [99]. Of note, the EZH2 inhibitor Tazemetostat may hold therapeutic potential for at least a subset of patients with advanced stage SWI/SNF complex-deficient solid tumors, including ES [100, 101].

Head and neck ES shows a high recurrence rate of 66.7% and a 5-year relapse-free survival of only 25.5% [21]. Localized primary ES of the oral cavity exhibits a more favorable prognosis following margin-negative surgical resection compared to metastatic intraoral ES.

## Conclusions

In summary, we expand the limited existing literature on ES of the oral cavity by reporting the clinicopathologic and immunophenotypic characteristics of 5 additional examples. Although rare, intraoral involvement by ES may occur either in the setting of primary or metastatic disease, with a strong site predilection for the tongue and gingiva of adolescents and young adults, and a markedly broad age range. The combined epithelioid, spindled, and, occasionally, rhabdoid cytomorphology of ES in conjunction with the observed aberrant immunoexpression of cytokeratins and EMA may cause a major diagnostic pitfall in oral biopsy specimens. A broad panel of epithelial, i.e., cytokeratins and p63/p40, and non-epithelial IHC markers, including SMARCB1, together with evaluation for surface dysplasia and a detailed clinical history, are necessary for exclusion of histologic mimics, establishing accurate diagnosis, and proper patient care.

## Data Availability

All available clinical, histopathologic and immunohistochemical data pertaining to the current case series are presented in the manuscript. Original files are available upon reasonable request from the corresponding author.
